# Age-specific gender differences in morphological parameters of intracranial aneurysms

**DOI:** 10.3389/fneur.2025.1480652

**Published:** 2025-04-08

**Authors:** Jianyao Mao, Yukui Li, Xin Gao, Zhangyu Li, Xi Chen, Guowei Tan, Zhanxiang Wang, Ping Zhong, Sifang Chen

**Affiliations:** ^1^Department of Neurosurgery, The First Affiliated Hospital of Xiamen University, School of Medicine, Xiamen University, Xiamen, China; ^2^The School of Clinical Medicine, Fujian Medical University, Fuzhou, China

**Keywords:** subarachnoid hemorrhage, intracranial aneurysms, gender difference, morphology, age

## Abstract

**Background:**

Gender differences are shown to exist in the incidence and outcome of subarachnoid hemorrhage as well as in the formation and progression of intracranial aneurysms. However, few studies investigated the gender difference in the morphology of intracranial aneurysms.

**Methods:**

A total of 308 consecutive patients with 346 intracranial aneurysms from 2014 to 2020 were retrospectively analyzed. Differences in 10 morphological parameters of intracranial aneurysms between males and females were compared. Continuous variables were expressed as the median [interquartile range (IQR)] and the differences were analyzed using the Mann-Whitney test. Categorical variables were expressed as numbers (frequencies) and the differences were analyzed using χ^2^ test. Moreover, subgroup analyses were performed according to age stratifications: ≥60 years, 50–59 years, and <50 years.

**Results:**

The median aspect ratio maximum [1.38, IQR (1.17–1.83) vs. 1.27, IQR (1.00–1.57)], median aspect ratio [1.29, IQR (1.00–1.76) vs. 1.18, IQR (0.93–1.54)], median bottleneck factor [1.17, IQR (1.00–1.47) vs. 1.03, IQR (0.86–1.27)], and median volume-to-ostium area ratio [5.67, IQR (2.85–9.03) vs. 3.86, IQR (1.94–7.48)] in males were significantly higher than those in females (all *P* < 0.01). Particularly, the differences in the above morphological parameters between genders were most prominent in patients aged ≥ 60 years, whereas no differences were found in patients aged < 50 years. There were no significant differences in other morphological parameters between males and females, including neck width, transverse, height, maximum, aneurysm volume, and height-width ratio.

**Conclusions:**

Gender difference existed in the morphology of intracranial aneurysms and was affected by age. The difference was prominent in patients aged ≥ 60 years, whereas no difference was found in patients aged < 50 years.

## Introduction

Subarachnoid hemorrhage (SAH) is a life-threatening stroke, largely leading to loss of many years of productive life owing to the relatively young age of those affected and high mortality (1). The rupture of an intracranial aneurysm is the cardinal cause, accounting for about 85% of cases (1, 2). Theoretically, risk factors for rupture of an unruptured aneurysm are similar to those for aneurysm formation and SAH (1). There is high agreement among neurosurgeons that the location and size are among pivotal risk factors for rupture of an intracranial aneurysm (3, 4). Moreover, in addition to size and location, several morphological parameters of intracranial aneurysms, including perpendicular height, aspect ratio, bottleneck factor, and aneurysm volume, have been shown to be associated with risk of rupture (5–8). Thus, aneurysm morphology could provide important implications in the clinical management of intracranial aneurysms.

Gender differences are suggested to exist not only in the incidence and outcome of SAH but also in the formation and progression of aneurysms (9). In a large consecutive series of ruptured intracranial aneurysms (3), the size and location varied considerably by gender, suggesting that gender differences existed in the location and size. However, no statistical method was applied to identify the significant differences in location and size between genders in the previous study (3). Moreover, one of the few studies suggested that females had a significant lower dome-to-neck ratio of intracranial aneurysms than males (10), but few studies investigated the detailed gender differences in aneurysm morphology so far (11). Furthermore, it was suggested that morphological parameters of posterior communicating artery aneurysms were associated with age (12). Therefore, we hypothesized that gender differences existed in the morphology of intracranial aneurysms and were affected by age. The purpose of this study was to explore the gender differences in morphological parameters of intracranial aneurysms, with a special focus on the gender differences stratified by age.

## Materials and methods

### Study population and data collection

The study population was from the First Affiliated Hospital of Xiamen University. The inclusion criteria were as follows: (1) Adult patients (≥18 years old) with saccular intracranial aneurysms admitted to the hospital between 2014 and 2020; (2) Ruptured and unruptured intracranial aneurysms which were repaired during hospitalization were both included; (3) The diagnosis of intracranial aneurysms was confirmed using CT angiography (CTA), 3-dimensional time-of-flight magnetic resonance angiography (3D-TOF-MRA), or digital subtraction angiography (DSA). The exclusion criteria were as follows: (1) The aneurysms belonged to traumatic aneurysms, feeding artery aneurysms to arteriovenous malformations (AVM), or fusiform aneurysms. (2) The patients whose aneurysms were treated prior to presentation. Demographic and clinical information, including age, gender, presence of multiple aneurysms, location of the aneurysms, and morphological parameters were recorded. This study was approved by the institutional review board under an expedited review and informed consent was waived for this retrospective study.

### Definition of location and morphological parameters

**Figure 1:**
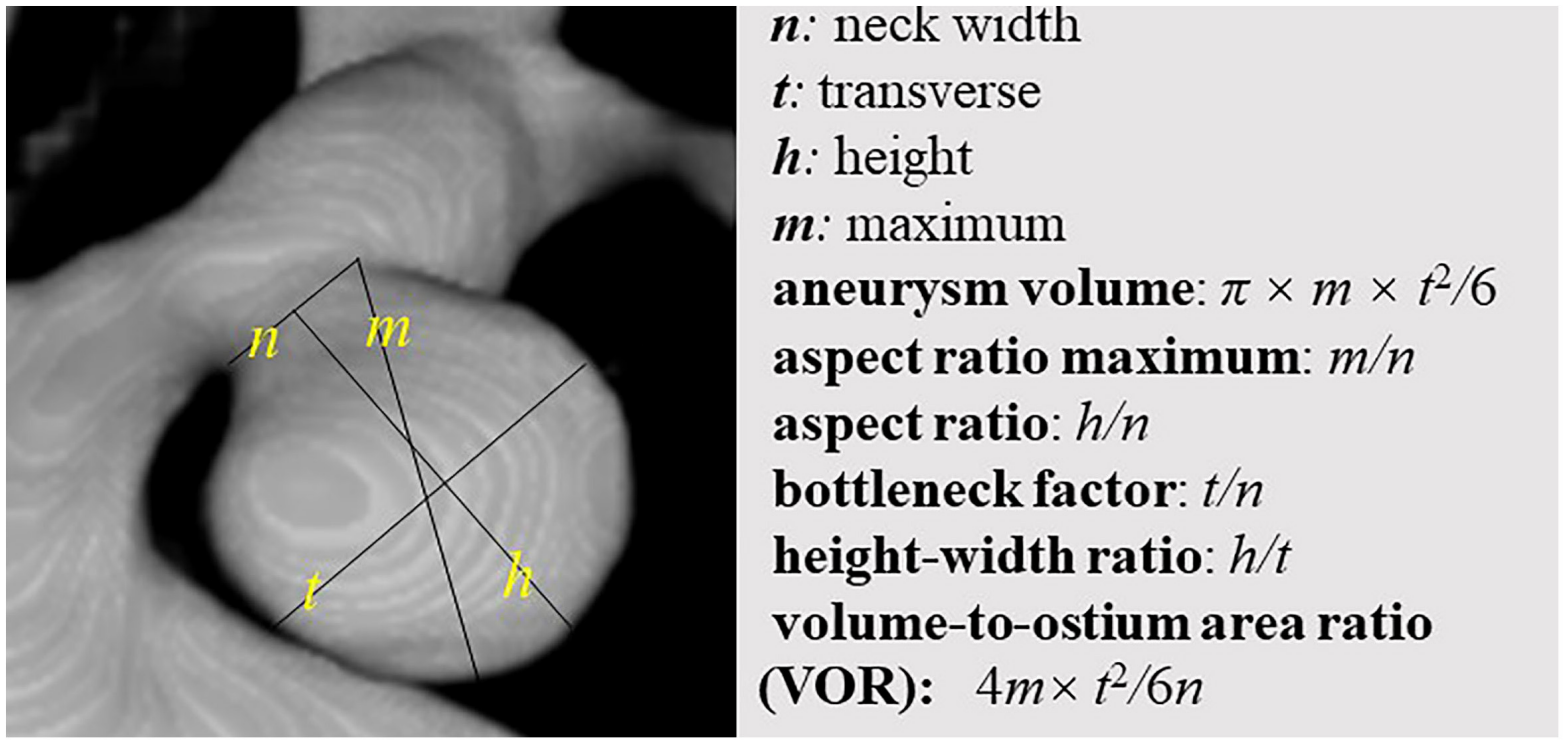
The detailed discrimination of 10 morphological parameters. The neck width (*n*), transverse (*t*), height (*h*), and maximum (*m*) were measured for each angiographic image of an intracranial aneurysm and used to calculate the morphological parameters as follows: (1) aneurysm volume: π × *m* × *t*^2^*/*6; (2) aspect ratio maximum: *m/n*; (3) aspect ratio: *h/n*; (4) bottleneck factor: *t/n*; (5) height-width ratio: *h/t*; (6) VOR: 4*m* × *t*^2^*/*6*n*^2^.

The locations of intracranial aneurysms were categorized as internal carotid posterior communication artery (ICPC), middle cerebral artery (MCA), anterior communication artery (ACoA), and others. The previous literature highlights a lot of morphological parameters associated with aneurysm growth and rupture risk, such as the ellipticity index, nonsphericity index, and size ratio, etc. (5–8). However, the results of Juvela and Korja (7) were derived from a long-term follow up study, and the results might be the most reliable. Thus, we thought that aneurysm volume, aspect ratio, bottleneck factor, height-width ratio, and volume-to-ostium area ratio (VOR) were pivotal morphological parameters for the assessment of intracranial aneurysms based on the previous study (7). Hence, the above morphological parameters were measured and calculated using the standard projection of 3-dimensional conventional angiograms in the present study. The neck width (*n*), transverse (*t*), height (*h*), and maximum (*m*) were measured for each angiographic image of an intracranial aneurysm and used to calculate the morphological parameters as follows (7): (1) aneurysm volume: π × *m* × *t*^2^*/*6; (2) aspect ratio maximum: *m/n*; (3) aspect ratio: *h/n*; (4) bottleneck factor: *t/n*; (5) height-width ratio: *h/t*; (6) VOR: 4*m* × *t*^2^*/*6*n*^2^. The detailed discrimination of the abovementioned morphological parameters is shown in Figure 1.

### Statistical analysis

The data were analyzed by SPSS statistic 22.0 (SPSS Inc., Chicago, USA). Initially, the Shapiro-Wilk test, stem-leaf plot, and normal P-P plot were used for the test of data distribution. Continuous variables were expressed as the median [interquartile range (IQR)] when they were non-parametric distribution, or mean (standard deviation) when they were parametric distribution. The Student's *t*-test was used for variables with parametric distribution and Mann-Whitney test for variables with the non-parametric distribution. Categorical values were expressed as number (frequencies), and the differences between the two groups were analyzed using χ^2^ test or Fisher's exact test. All statistical significance was defined as *P* < 0.05.

Initially, the differences in age, presence of multiple aneurysms, location of the aneurysms, and morphological parameters were compared between males and females. Moreover, although the majority of the overall aneurysm population is females, the prevalence was reported to be equivalent between males and females among adults younger than 50 years (13). Furthermore, 60 years old was suggested to be as the boundary to differentiate the risk of growth of unruptured intracranial aneurysms (14). Therefore, subgroup analyses of differences in location and morphological parameters of intracranial aneurysms between gender were further performed according to the age stratification: ≥60 years, 50–59 years, and <50 years. Additionally, the association between age and morphological parameters was evaluated as well. Considering gender could affect the morphological parameters, morphological parameters of intracranial aneurysms between patients aged ≥ 50 years and patients aged < 50 years were compared separately in males and females.

## Results

### Basic demographic and clinical characteristics of 308 patients with intracranial aneurysms

**Figure 2:**
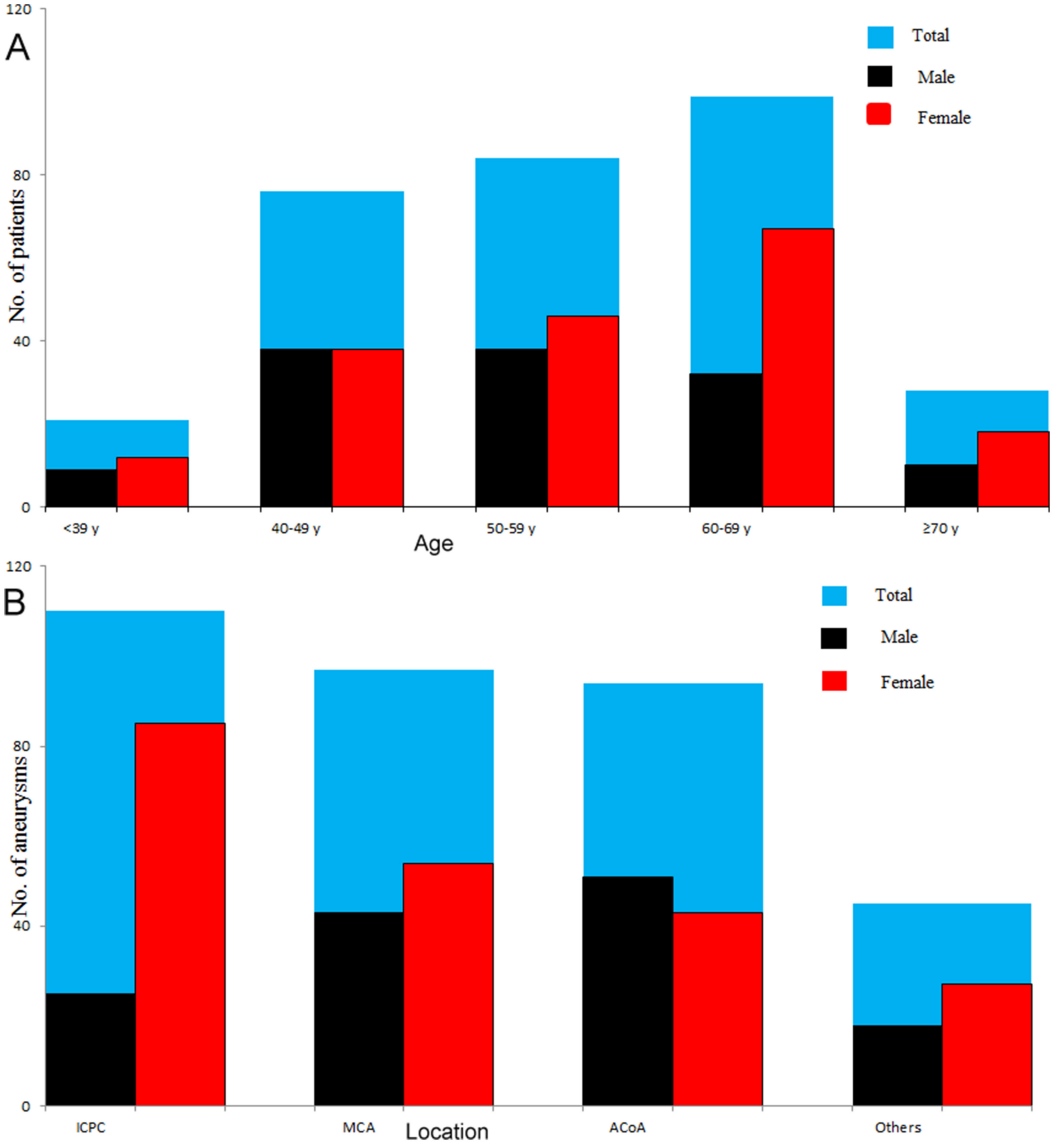
Distributions of age and location of males and females with intracranial aneurysms. The number was equivalent between males and females among patients younger than 50 years, whereas females had a significantly larger number than males in patients aged ≥ 50 years, especially in patients aged ≥ 60 years **(A)**. The top three locations of intracranial aneurysms were ICPC (31.79%), MCA (28.03%), and ACoA (27.17%), accounting for 86.99% of all intracranial aneurysms **(B)**. ICPC, internal carotid posterior communication artery; MCA, middle cerebral artery; ACoA, anterior communication artery.

The study included 308 patients with 346 intracranial aneurysms, of which 127 (137 aneurysms) were males, and 181 (209 aneurysms) were females. The average age was 55.68 ± 11.34 years, in which males and females were 54.29 ± 11.35 years and 56.66 ± 11.26 years, respectively. In this case series, 10.71% of the patients presented multiple aneurysms, and the majority (89.02%) of the aneurysms were <10 mm. The distributions of age and location of males and females with intracranial aneurysms are shown in Figure 2. The female to male ratio was approximately one among patients younger than 50 years, but the ratio was far more than one among patients aged ≥ 50 years. The top three locations of intracranial aneurysms were ICPC (31.79%), MCA (28.03%), and ACoA (27.17%), accounting for 86.99% of all intracranial aneurysms.

### Comparisons of age, location, and morphological parameters of aneurysms between males and females

The comparisons of age, location, and morphological parameters of aneurysms between males and females are shown in Table 1. The percentage of patients aged ≥ 60 years in females was significantly higher than that in males (46.96 vs. 30.07%, *P* = 0.047). There was a significant difference in the distribution of the location of aneurysms between genders (*P* = 0.000), with a higher percentage of ICPC aneurysm in females (40.67 vs. 18.25%) and a higher percentage of ACoA aneurysm in males (37.22 vs. 20.57%). The median aspect ratio maximum [1.38, IQR (1.17–1.83) vs. 1.27, IQR (1.00–1.57)], aspect ratio [1.29, IQR (1.00–1.76) vs. 1.18, IQR (0.93–1.54)], bottleneck factor [1.17, IQR (1.00–1.47) vs. 1.03, IQR (0.86–1.27)], and VOR [5.67, IQR (2.85–9.03) vs. 3.86, IQR (1.94–7.48)] in males were significantly higher than those in females (all *P* < 0.01). There were no significant differences in other morphological parameters between males and females, including neck width, transverse, height, maximum, aneurysm volume, and height-width ratio.

**Table 1:** Comparisons of age, location, and morphological parameters of aneurysms between males and females.

**Variables**	**All patients (*n*_1_ = 308, *n*_2_ = 346)**	**Males (*n*_1_ = 127, *n*_2_ = 137)**	**Females (*n*_1_ = 181, *n*_2_ = 209)**	***t/Z*/*χ^2^***	***P***
Age (y)	55.68 ± 11.34	54.29 ± 11.35	56.66 ± 11.26	1.809	0.071
≥60	127 (41.23)	42 (30.07)	85 (46.96)	6.135	0.047^*^
50–59	84 (27.27)	38 (29.92)	46 (25.41)		
<50	97 (31.50)	47 (37.01)	50 (27.63)		
Multiple aneurysms (*n*, %)	33 (10.71)	10 (7.87)	23 (12.71)	1.822	0.177
**Size (mm)**					
≥10	38 (10.98)	17 (12.41)	21 (10.05)	0.491	0.782
5–10	173 (50.00)	68 (49.64)	105 (50.24)		
<5	135 (39.02)	52 (37.95)	83 (39.71)		
**Location (** ***n*** **, %)**					
AcoA	94 (27.17)	51 (37.22)	43 (20.57)	22.445	0.000^&^
MCA	97 (28.03)	43 (31.39)	54 (25.84)		
ICPC	110 (31.79)	25 (18.25)	85 (40.67)		
Others	45 (13.01)	18 (13.14)	27 (12.92)		
Unruptured aneurysms (*n*, %)	40 (11.56)	11 (8.03)	29 (13.88)	2.767	0.096
**Morphology**					
Neck width (mm)	4.20 (3.10–5.20)	4.00 (3.00-5.25)	4.20 (3.30-5.20)	−1.019	0.308
Height (mm)	5.10 (4.20–7.60)	5.00 (3.90–7.55)	5.10 (3.45–6.95)	−1.177	0.239
Transverse (mm)	4.40 (3.10–6.40)	4.70 (3.35–6.55)	4.10 (3.00–6.25)	−1.514	0.130
Maximum (mm)	5.75 (4.20–7.60)	5.80 (4.35–8.25)	5.70 (4.20–7.45)	−0.915	0.360
Aneurysm volume (mm^3^)	57.42 (22.49–155.97)	68.66 (25.27–179.50)	51.67 (21.22–143.72)	−1.495	0.135
Aspect ratio maximum	1.31 (1.08–1.68)	1.38 (1.17–1.83)	1.27 (1.00–1.57)	−3.415	0.001^&^
Aspect ratio	1.23(0.96–1.60)	1.29 (1.00–1.76)	1.18 (0.93–1.54)	−2.802	0.005^&^
Bottleneck factor	1.07 (0.90–1.31)	1.17 (1.00–1.47)	1.03 (0.86–1.27)	−4.317	0.000^&^
Height-width ratio	1.12 (0.90–1.46)	1.11 (0.87–1.50)	1.03 (0.86–1.27)	−0.281	0.779
VOR	4.55 (2.31–8.23)	5.67 (2.85–9.03)	3.86 (1.94–7.48)	−3.322	0.001^&^

ICPC, internal carotid posterior communication artery; MCA, middle cerebral artery; ACoA, anterior communication artery; VOR, volume-to-ostium area ratio.

* Denoted *P* < 0.05 between males and females.

& Denoted *P* < 0.01 between males and females.

### Comparisons of the location of intracranial aneurysms between males and females stratified by age

The Comparisons of the location of intracranial aneurysms between males and females according to age stratification are presented in Table 2. In the subgroups of patients aged ≥ 60 and <50 years, no significant differences in the distributions of the location of intracranial aneurysms were observed between genders. However, there was a significant difference in the distribution of the location of intracranial aneurysms between males and females when restricted in the patients aged 50–59 years (*P* = 0.000). In this subgroup, the percentage of ICPC aneurysm in females was significantly higher than that in males (52.46 vs. 7.14%). In contrast, the percentages of AcoA and MCA aneurysms in males were significantly higher than those in females (45.23 vs. 16.39%, 33.33 vs. 18.03%).

**Table 2:** Comparisons of the location of intracranial aneurysms between males and females stratified by age.

**Age (M/F)**	**Location**	**Males**	**Females**	***χ^2^***	***P***
≥60 y (44/95)	AcoA	11 (25.00)	20 (21.05)	3.265	0.351
	MCA	17 (38.64)	27 (28.42)		
	ICPC	11 (25.00)	38 (40.00)		
	Others	5 (11.36)	10 (10.53)		
50–59 y (42/61)	AcoA	19 (45.23)	10 (16.39)	24.807	0.000^&^
	MCA	14 (33.33)	11 (18.03)		
	ICPC	3 (7.14)	32 (52.46)		
	Others	6 (14.30)	8 (13.12)		
<50 y (51/53)	AcoA	21 (41.18)	13 (24.53)	3.282	0.350
	MCA	12 (23.53)	16 (30.19)		
	ICPC	11 (21.57)	15 (28.30)		
	Others	7 (13.72)	9 (16.98)		

ICPC, internal carotid posterior communication artery; MCA, middle cerebral artery; ACoA, anterior communication artery.

& Denoted *P* < 0.01 between males and females.

### Comparisons of morphological parameters of intracranial aneurysms between males and females stratified by age

The comparisons of morphological parameters of intracranial aneurysms between males and females according to age stratification are shown in Table 3. In the subgroup of patients aged ≥ 60 years, the median aneurysm volume [137.66, IQR (43.80–281.88) mm^3^ vs. 58.06, IQR (22.44–152.56) mm^3^], aspect ratio maximum [1.46, IQR (1.28–1.84) vs. 1.26, IQR (1.00–1.56)], aspect ratio [1.34, IQR (1.10–1.74) vs. 1.15, IQR (0.92–1.49)], bottleneck factor [1.21, IQR (1.01–1.56) vs. 1.00, IQR (0.86–1.24)], and VOR [6.95, IQR (4.55–13.77) vs. 3.86, IQR (2.08–7.54)] in males were significantly higher than those in females (all *P* < 0.01). However, only aspect ratio maximum and aspect ratio showed significant differences between males and females aged 50–59 years. Of note, no significant differences in the morphological parameters were observed between males and females younger than 50 years.

**Table 3:** Comparisons of morphological parameters of intracranial aneurysms between males and females stratified by age.

**Age (M/F)**	**Morphology**	**Males**	**Females**	***Z***	***P***
≥60 y (44/95)	Aneurysm volume (mm^3^)	137.66 (43.80–281.88)	58.06 (22.44–152.56)	−1.972	0.049^*^
	Aspect ratio maximum	1.46 (1.28–1.84)	1.26 (1.00–1.56)	−3.525	0.000^&^
	Aspect ratio	1.34 (1.10–1.74)	1.15 (0.92–1.49)	−2.787	0.005^&^
	Bottleneck factor	1.21 (1.01–1.56)	1.00 (0.86–1.24)	−3.784	0.000^&^
	VOR	6.95 (4.55–13.77)	3.86 (2.08–7.54)	−3.303	0.001^&^
50–59 y (42/61)	Aneurysm volume (mm^3^)	62.27 (23.52–165.87)	47.69 (20.33–142.07)	−0.507	0.612
	Aspect ratio maximum	1.56 (1.09–1.97)	1.25 (1.00–1.56)	−2.149	0.032^*^
	Aspect ratio	1.51 (0.92–1.96)	1.16 (0.84–1.52)	−1.976	0.048^*^
	Bottleneck factor	1.16 (0.99–1.41)	1.06 (0.80–1.27)	−1.775	0.076
	VOR	5.69 (2.64–8.63)	4.00 (1.85–7.63)	−1.590	0.112
<50 y (51/53)	Aneurysm volume (mm^3^)	50.24 (23.13–128.52)	36.90 (16.75–116.80)	−0.631	0.528
	Aspect ratio maximum	1.26 (1.15–1.74)	1.37 (1.07–1.71)	−0.075	0.940
	Aspect ratio	1.24 (1.00–1.57)	1.25 (1.00–1.57)	−0.072	0.943
	Bottleneck factor	1.13 (1.00–1.38)	1.03 (0.86–1.25)	−1.811	0.070
	VOR	4.05 (2.60–8.77)	3.24 (1.88–7.06)	−0.962	0.336

Neck width, transverse, height, maximum, and height-width ratio were not significantly different between genders in all age subgroups.

VOR, volume-to-ostium area ratio.

* Denoted *P* < 0.05 between males and females.

& Denoted *P* < 0.01 between males and females.

### Comparisons of morphological parameters of intracranial aneurysms between patients aged ≥ 50 years and patients aged < 50 years stratified by gender

The comparisons of morphological parameters of intracranial aneurysms between patients aged ≥ 50 years and patients aged <50 years stratified by gender are presented in Table 4. Males aged ≥ 50 years had a larger maximum, aneurysm volume, and aspect ratio maximum compared with those aged < 50 years. However, no significant difference in aneurysm morphology was found between females aged ≥ 50 years and those aged < 50 years.

**Table 4:** Comparisons of morphological parameters of intracranial aneurysms between patients aged ≥ 50 years and patients aged < 50 years stratified by gender.

**Gender (≥50/<50)**	**Morphology**	**≥50 years**	**<50 years**	***Z***	***P***
Males (86/51)	Maximum (mm)	6.55 (4.50–9.00)	5.10 (4.00–6.60)	−2.289	0.022^*^
	Aneurysm volume (mm^3^)	95.32 (35.18–222.52)	50.24 (23.13–128.52)	−2.033	0.042^*^
	Aspect ratio maximum	1.53 (1.20–1.96)	1.26 (1.15–1.74)	−1.968	0.049^*^
Females (156/53)	Maximum (mm)	5.90 (4.23–7.60)	5.30 (3.90–7.30)	−1.158	0.247
	Aneurysm volume (mm^3^)	56.20 (21.78–149.58)	36.90 (16.76–116.81)	−1.300	0.194
	Aspect ratio maximum	1.26 (1.00–1.55)	1.37 (1.07–1.71)	−1.354	0.176

Neck width, transverse, height, aspect ratio, bottleneck factor, height-width ratio, and volume-to-ostium area ratio were not significantly different between patients aged ≥ 50 years and patients aged < 50 years in both male and female subgroups.

* Denoted *P* < 0.05 between patients aged ≥ 50 years and those aged < 50 years.

## Discussion

The main findings of our study are, first, that gender differences existed in the morphology of intracranial aneurysms. More specifically, males had remarkably larger aneurysm volume, aspect ratio maximum, aspect ratio, bottleneck factor, and VOR compared with females. Second, the gender difference in aneurysm morphology was affected by age. The gender difference was the most prominent in patients aged ≥ 60 years, whereas it was not significant in patients younger than 50 years. To the best of our knowledge, this is the first study to report the age-specific gender difference in the morphology of intracranial aneurysms.

Compared with published large case series (3, 15, 16), this study population had similar basic demographic and clinical characteristics, including mean age, female to male ratio, the incidence of multiple aneurysms, the incidence of the small aneurysm (size < 10 mm), and distribution of aneurysm location. For instance, the proportion of aneurysms smaller than 10 mm was previously reported to be 78–94% (3, 15), which was similar to our result (89.02%). Additionally, both the previous studies (3, 15, 16) and our results demonstrated that ICPC, MCA, and ACoA were the three most common locations of intracranial aneurysms. Accordingly, the study population could be thought to represent the overall aneurysm distribution in our local population.

In clinical practice, since females have a higher prevalence of intracranial aneurysms than males (17), gender difference is a remarkable clinical characteristic in patients with intracranial aneurysms. In this study, gender difference was also observed in aneurysm morphology, which is in keeping with those of a published report on the influence of gender on morphological parameters of intracranial aneurysms (11). However, Lin et al. (11) reported that the gender differences in aneurysm morphology were restricted in size, height, and size ratios, while the differences in other morphological parameters were not observed. This discrepancy might be attributed to the gender ratio of the study population, in which the female to male ratio was <1 in Lin et al.'s study but far more than one in our study as well as previous literature (3, 15, 16). In line with previous study (10), we found that males had significantly larger aspect ratio maximum, aspect ratio, bottleneck factor, and VOR than females. The gender differences in aneurysm morphology may be explained by the distinct degrees of inflammation in aneurysm formation between genders. First, the hemodynamic and inflammatory mechanisms are suggested to be the putative pathogenesis of intracranial aneurysms (18). In particular, the inflammation cascade is critical to wall remodeling, which is a major process in the formation, growth, and rupture of intracranial aneurysms (19). Previously, the gender difference in wall remodeling induced by inflammation was observed in a rat saccular intracranial model (20). In the animal study (20), vessel wall macrophage content was significantly higher in intracranial aneurysms of male rats at 28 days than in female rats, suggesting that males might have a more advanced degree of local aneurysmal wall inflammation than females. Second, evidence in animals and humans indicated that aspirin, a non-steroidal anti-inflammatory drug, decreased the risk of aneurysm rupture more significantly in males than in females (21). Third, aneurysmal wall enhancement (AWE) on high-resolution vessel wall magnetic resonance imaging, which could reflect aneurysmal wall inflammation, was suggested to be associated with an irregular shape, a higher aspect ratio, and a higher bottleneck factor (22, 23). Thus, this study supports evidence from previous observations. However, the pathophysiological basis accounting for these dissimilarities remains unclear, and therefore further research is recommended to be undertaken to investigate the difference in inflammation patterns of aneurysm formation between genders.

Interestingly, we found that gender difference in aneurysm morphology was significant in patients aged ≥ 50 years, especially in patients aged ≥ 60 years. The reason for this is not clear but it may have something to do with the age-related difference in aneurysmal wall inflammation. On the one hand, a strong relationship between age and aneurysmal wall inflammation has been reported in the literature (24). For instance, it was suggested that older age was independently associated with increased aneurysmal wall enhancement on MRI (24), indicating that older patients with intracranial aneurysms might present a higher degree of aneurysmal wall inflammation. On the other hand, our result further showed that older males had a larger maximum, aneurysm volume, and aspect ratio maximum, whereas no difference in aneurysm morphology was found in females between different age groups. In this situation, it makes sense that the gender difference in aneurysm morphology would be more significant in older patients. In addition to aneurysm morphology, we found that gender difference in location of aneurysms was affected by age, which was consistent with Horiuchi et al.'s study (25). Surprisingly, only patients aged 50–59 years showed a statistically significant difference in aneurysm location between genders when stratified by age in this study. It is difficult to explain this result, but it might be related to a rapid estrogen reduction in females during this period. A further study is therefore suggested to verify this hypothesis.

Our findings could give further support to the previously proposed pathophysiological features of intracranial aneurysms, providing important implications to future studies that can discern the critical steps in the pathogenesis of intracranial aneurysms. Importantly, since aneurysm morphology plays a crucial role in outcomes of intracranial aneurysms, future studies on the relevant subjects are suggested to take the age-specific gender differences seriously into account.

## Limitations

Despite the intriguing findings of this study, several important limitations should be taken into account. First, our study is single-centered research with a relatively small sample size, and it may be underpowered to detect a significant difference in location and morphology of intracranial aneurysms between genders stratified by age. Second, over 85% of the aneurysms were ruptured in this study, and whether gender differences in location and morphology of intracranial aneurysms are attributed to rupture remains unknown. Further studies, which take these variables into account, will need to be undertaken. Last but not least, gender differences in aneurysm morphology were interpreted with aneurysmal wall inflammation in this study. Although this interpretation is supported by a large number of published articles, we could not provide direct evidence to validate this inference. Accordingly, the pathophysiological basis accounting for these dissimilarities remains to be established. Future studies on the current topic are therefore recommended.

## Conclusion

In summary, gender differences existed in the morphology of intracranial aneurysms and were affected by age. Males had a larger aspect ratio maximum, aspect ratio, bottleneck factor, and VOR compared with females. Particularly, gender differences in aneurysm morphology were prominent in patients aged ≥ 60 years, whereas it was not significant in patients younger than 50 years. Previously proposed inflammatory and hormonal theories behind the pathogenesis of intracranial aneurysms seem like plausible mechanisms to explain the differences we found.

## Data Availability

The raw data supporting the conclusions of this article will be made available by the authors, without undue reservation.
